# The Interrelationships of Three Change of Direction Ability Tests in Elite Female Volleyball Players: An Exploratory Study

**DOI:** 10.3390/sports13100339

**Published:** 2025-10-02

**Authors:** Will C. Wright, Elroy J. Aguiar, Lee J. Winchester, Michael V. Fedewa, Andrew D. Fields, Michael R. Esco

**Affiliations:** 1 Department of Kinesiology, The University of Alabama, Tuscaloosa, AL 35487, USAmvfedewa@ua.edu (M.V.F.); adfields1@crimson.ua.edu (A.D.F.); 2 Department of Athletics, Jacksonville State University, Jacksonville, AL 36265, USA

**Keywords:** volleyball, agility testing, change of direction, elite athlete, female athlete

## Abstract

Background: The purpose of this exploratory study is to evaluate the relationships between commonly used change of direction COD tests (*t*-test, L-drill, and 5-10-5 shuttle) in Division I female volleyball players. Methods: Sixteen Division I female volleyball players (age = 19.4 ± 1.4 years, height = 176.2 ± 10.6 cm, weight = 71.5 ± 11.1 kg) participated in this study and completed three trials of the *t*-test, L-drill, and 5-10-5 shuttle in a randomized order. The best time for each test was recorded and analyzed. Pearson product correlations were used to determine the interrelationships between each of the three COD tests. Additionally, a composite score was created by summing the best performances of the three drills. The relationship between each test and the composite score was also established via Spearman rank correlations. Results: The mean ± standard deviation for time to complete each test was 10.9 ± 0.7 s for the *t*-test, 8.6 ± 0.3 s for the L-drill, and 4.9 ± 0.2 s for the 5-10-5. Statistically significant correlations were shown between the *t*-test and L-Drill (*r* = 0.89, *p* < 0.001,), between the L-drill and 5-10-5 Shuttle (*r* = 0.91, *p* < 0.001), and between the 5-10-5 Shuttle and *t*-test (*r* = 0.83, *p* < 0.001). In addition, each COD test significantly correlated with the composite score (ρ = 0.92–0.95). Conclusion: The high degree of agreement between the three COD tests suggests that any one of them could be utilized for testing COD ability in female volleyball players.

## 1. Introduction

Change-of-direction (COD) ability refers to the capacity to alter movement direction, speed, or mode, and is a critical component of optimal performance in many sports [1]. In team-based sports, COD ability is especially vital, as players must frequently execute explosive, multidirectional movements within shortened timeframes [2,3,4,5]. Furthermore, movements during play often involve dynamic transitions, including accelerations, decelerations, turns, and curvilinear running [3,4,5]. In volleyball, these repeated short-duration bursts of movement often determine rally outcomes [2]. Skills such as blocking, digging, and counterattacking rely heavily on rapid directional shifts, making COD testing a practical tool for guiding training and monitoring performance in this sport [2]. Given its central role in volleyball performance, a variety of methods have been developed to assess COD ability.

Among the most commonly used assessments are the *t*-test, L-drill, and 5-10-5 shuttle (also known as the pro agility or 20-yard shuttle). While all three tests aim to measure COD ability, they differ in structure and movement demands. The methodological differences may place distinct neuromuscular and biomechanical demands on the athlete. Although all three tests are widely used in athletic, research, and recreational settings and have demonstrated high test–retest reliability [6,7,8,9], their level of agreement and interchangeability remains unclear. Some studies suggest COD tests assess different components of COD ability [10,11], whereas others report significant inter-test correlations, suggesting overlap in the attributes they measure [9].

While these results support the general utility of each test in diverse populations, further investigation is warranted within sport-specific contexts. Strength and conditioning programs are often tailored to enhance the specific physical and cognitive skills that align with the demands of a given sport. As athletes become more specialized, they may demonstrate superior performance in COD ability that more closely resemble sport-specific movement patterns. This is particularly critical in volleyball due to the sport’s dynamic and high-speed nature. Athletes must repeatedly execute quick, multi-directional movements in response to unpredictable gameplay [12,13,14]. Additionally, although COD has been studied extensively in male athletes and in field-based sports such as soccer and football [1,8], relatively little research has examined these constructs in female volleyball players. Given the sex- and sport-specific biomechanical and neuromuscular demands that influence COD ability [15], studies in this population are needed to better inform practice. Moreover, COD is not the same as, but rather, foundational to agility, which involves more perceptual and decision-making responses to an external stimulus [16]. Thus, testing COD in a controlled environment isolates the physical ability to decelerate, change direction, and reaccelerate in a pre-planned context [14]. These considerations highlight the importance of examining COD test interrelationships specifically within elite female volleyball athletes.

Therefore, identifying the inter-correlations across COD tests could help coaches and practitioners select the most appropriate tests for evaluating performance and monitoring training progress. Accordingly, the objective of this study was to explore the interrelationships among three commonly used COD tests (*t*-test, L-drill, and 5-10-5 shuttle) in Division I female volleyball players. We hypothesized that significant positive correlations would be observed among all three COD tests, thereby supporting their shared utility for evaluating COD ability in this population.

## 2. Materials and Methods

### 2.1. Subjects

Sixteen competitive female athletes from a National Collegiate Athletic Association Division 1 volleyball team participated in this exploratory study. All participants were considered physically healthy and were cleared for sport participation via the athletic department’s team physician. Data collection for each participant occurred within the volleyball arena on a single day of the week during regular scheduled training sessions. Participants were told to refrain from consuming depressants (e.g., alcohol), maintain regular dietary habits, and avoid excess physical activity outside of their normal athletic requirements for 24 h prior to study. Participants completed a 24 h history questionnaire to confirm their compliance. The University’s Institutional Review Board (IRB) approved the study protocol, and all participants provided written informed consent.

### 2.2. Procedures

Following the completion of the previously mentioned documents, anthropometric data was collected. Body height was measured using a manual stadiometer to the nearest 0.1 cm (HM 200P, Charder Electronic Co., Ltd., Taichung, Taiwan). Body weight was measured to the nearest 0.1 kg using a digital scale (RD-545PRO, Tanita Corporation, Tokyo, Japan).

The participants were then required to perform three commonly utilized COD tests (*t*-test, L-drill and 20-yard shuttle) in a random order. These tests have previously demonstrated high test–retest reliability in team-sport athletes, with reported intraclass correlation coefficients (ICCs) of 0.95 for the *t*-test, 0.94 for the L-drill, and 0.90 for the 5-10-5 shuttle [9]. The random order of testing was established before the study began in Microsoft Excel using the function RANDBETWEEN with the lower limit set at 1 and an upper limit at 3. The tests were assigned the following codes: *t*-test = 1; L-drill = 2; 5-10-5 Shuttle = 3. After the order was assigned to the participant, they completed a standardized dynamic warm-up. The warm-up consisted of a three-minute jog, dynamic movement drills (high knees, straight leg march, walking quad stretch), calisthenics (squats and lunges), as well as low intensity plyometrics (skipping, and lateral jumps).

After the completion of the warm-up, the participants had a five-minute recovery period before beginning the first COD test. For each COD test, the participants performed two familiarization trials at a moderate-intensity pace. Then, the subjects performed three recorded attempts of each COD test at maximal effort. The participants were given 1–2 min of rest between each attempt and approximately 10 min of rest between each COD test. Each COD test was performed on a volleyball court and each participant wore their competition footwear during the testing. Times for all three COD tests were measured via the same timing gate system (TCi system, Brower Timing Systems, Draper, UT, USA). During testing, there were two Certified Strength and Conditioning Specialists present to ensure that the participants maintained proper technique. To successfully complete each test, the participant had to touch the cone at each change in direction point. In the event of an unsuccessful attempt, the participant was permitted one additional attempt. If the participant experienced an egregious error such as tripping or falling, they were also given another attempt (*n* = 0).

**Figure 1:**
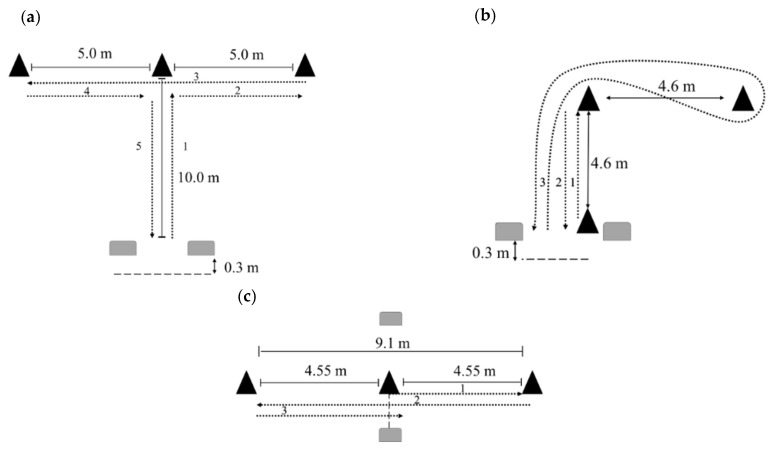
Visual diagrams of the setups and execution of the *t*-test (**a**), L-drill (**b**), and 5-10-5 test (**c**).

The *t*-test protocol followed the descriptions provided by Semenick [17] and Stewart et al. [9] and is illustrated in Figure 1a. The test setup consists of four cones arranged in a “T” shape. Cone A (the starting cone) is positioned at the base of the “T.” Cone B is placed 9.1 m directly ahead of Cone A. From Cone B, two additional cones (Cones C and D) are positioned 4.55 m to the left (Cone C) and right (Cone D), respectively, forming the top of the “T.” Subjects began just behind the timing gate at Cone A. Timing began as soon as they sprinted 9.1 m forward to Cone B and touched it. From there, they performed a lateral shuffle 4.55 m to the left to Cone C, touched the cone, then shuffled 9.1 m across to Cone D and touched it as well. Finally, they shuffled 4.55 m back to the middle to touch Cone B. They concluded the test by backpedaling 9.1 m through the timing gate at Cone A, which stopped the timer.

The L-drill protocol followed the design proposed by Stewart et al. [9] and is presented in Figure 1b. The drill setup consists of three cones arranged in an “L” shape. Cone 1 (the starting cone) is placed at the base of the “L.” Cone 2 is positioned 4.6 m straight ahead of Cone 1, and Cone 3 is placed 4.6 m to the right of Cone 2, forming a 90° angle between all three cones. This configuration creates an inverted “L” layout that challenges the athlete’s ability to accelerate, decelerate, and execute sharp and curvilinear directional changes. Timing gates were placed at the start/finish line (Cone 1), with timing beginning as the athlete broke the beam at the start and ending once they re-crossed the beam upon finishing. Subjects were instructed to begin at Cone 1 and initiate the test with a 4.6 m sprint to Cone 2, where they touched the cone and immediately returned to Cone 1. After touching Cone 1, they performed a 180° turn and sprinted back toward Cone 2, continuing around it with a 90° turn toward Cone 3. The participant then looped around Cone 3 with a 180° turn and sprinted 9.2 m back through the starting line (Cone 1) to complete the test.

The 5-10-5 shuttle test followed the design described by McGuigan [18] and is also illustrated in Figure 1c. The setup includes three cones placed in a straight line, with the middle cone serving as both the starting and finishing point. The two outer cones are positioned 4.55 m (5 yards) to the left and right of the center cone, respectively, creating a total distance of 9.1 m (10 yards) between the two end cones. Subjects began the test by straddling the center cone, with timing gates set up at this midpoint to serve as both the start and finish line. Timing began as the participant turned 90° and sprinted 4.55 m to one side to touch the first cone. They then turned sharply 180° and sprinted 9.1 m in the opposite direction to touch the second cone. After touching the second cone, they executed a final 180° turn and sprinted 4.55 m back through the timing gates at the center cone, which stopped the timer.

### 2.3. Statistical Analysis

Statistical analyses were performed using SPSS software (version 28.0, IBM Corporation, New York, NY, USA). Means and standard deviations (SD) were determined for all the studied variables. Normality was confirmed by comparing histograms to a normal curve which was followed with the Shapiro–Wilk test that displayed a *p* value greater than 0.05 [19]. Pearson correlation coefficients were used to determine the relationships between the three tests. Using the scale proposed by Hopkins [20], correlation values between 0 and 0.10 were considered “trivial”, 0.10 to 0.30 “small”, 0.30 to 0.50 “moderate”, 0.50 to 0.70 “large”, 0.70 to 0.90 “very large”, and 0.90 to 1.00 “nearly perfect”. A composite COD score was also calculated for each participant by summing the best score for each test. A Spearman rank correlation was then utilized to determine the relationship between each COD test and the composite score. Statistical significance was set at *p* < 0.05.

## 3. Results

All sixteen participants successfully completed the testing protocol. Descriptive statistics for the participants are presented in Table 1.

**Figure 2:**
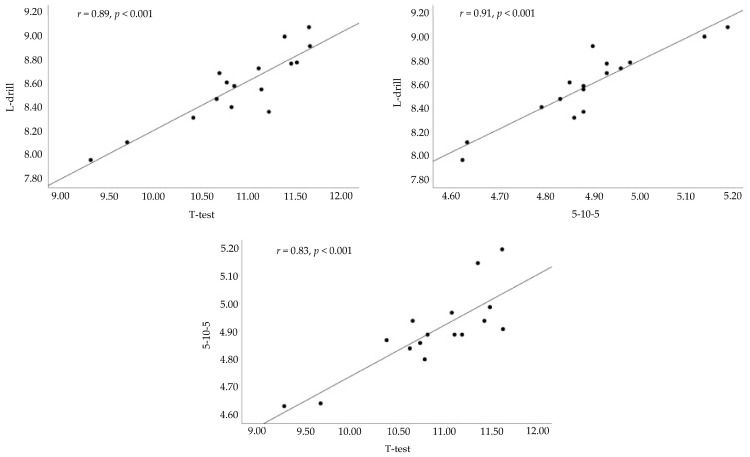
Scatterplot diagrams displaying correlations between change in direction tests. The unit of measurement on each axis is seconds.

The mean scores, represented as mean ± SD, for the COD tests are also shown in Table 1. Pearson correlations revealed significant coefficients between the *t*-test and L-drill (*r* = 0.89, *p* < 0.001, “very large”), between the L-drill and 5-10-5 Shuttle (*r* = 0.91, *p* < 0.001, “near perfect”), and between the 5-10-5 Shuttle and *t*-test (*r* = 0.83, *p* < 0.001, “very large”). Scatter plots illustrating these relationships are presented in Figure 2. A composite COD score was then calculated by summing the scores of each test for each participant and displayed a mean (±SD) or 24.37 ± 1.08 s. The Spearman rank correlation revealed that the *t*-test (ρ = 0.95, *p* < 0.001), L-drill (ρ = 0.92, *p* < 0.001), and 5-10-5 (ρ = 0.92, *p* < 0.001) each displayed relationships with the composite score.

## 4. Discussion

The purpose of this exploratory study was to examine the interrelationships among three commonly used COD tests (the *t*-test, the L-drill, and the 5-10-5 Shuttle) in Division I female volleyball players. The results demonstrated very large to near perfect correlations (r = 0.83–0.91, *p* < 0.001) among all three tests, indicating that they assess highly overlapping aspects of COD ability in this population. From a practical perspective, this suggests that coaches and sport scientists do not need to administer multiple COD assessments to obtain valid information. Instead, any one of the three tests can be selected based on available time, space, or equipment, reducing testing burden while still providing reliable insight into athlete performance. These findings are particularly useful in volleyball settings where time for physical testing is often limited due to the technical, tactical, and competitive demands of the sport.

Previous studies have reported significant correlations among COD tests, though most have involved non-athletic or mixed-sport samples of primarily male participants [8,10,21]. For instance, strong associations between the *t*-test and other COD tests (e.g., 5-0-5, Edgren Side Step Test, Illinois agility test) have been shown in male soccer players [8] and servicemembers [21]. Similarly to the current study, Stewart et al. [9] found significant correlations between the *t*-test, L-drill, and 5-10-5, as well as the 5-0-5, and Illinois agility tests, in a sample of recreationally active adult men and women, with correlation coefficients ranging from 0.84 to 0.89. The present study extends this body of work by confirming very large to near perfect interrelationships among the *t*-test, 5-10-5, and L-drill specifically in Division I female volleyball players, a group with distinct sport-specific movement demands. The findings support the notion that these tests capture overlapping COD attributes yet still emphasize unique directional and biomechanical patterns. As such, their strong interrelationships do not necessarily negate their individual value. Instead, they suggest that COD tests could potentially be used interchangeably, yet their utility may be context-dependent, particularly in sports requiring repeated multidirectional movements [8,9,10,21].

The very large to near perfect correlations observed among the three COD tests may be attributed to several overlapping physiological, biomechanical, and sport-specific demands. All three tests were completed in under 11 s, a similar duration of ball-in-play sequences in volleyball. This similarity suggests a predominant reliance on the immediate anaerobic energy system during these tests, which predominates during high-intensity activities lasting up to 10 s [14]. Such energy system alignment likely enhances performance transfer between COD tests and sport-specific tasks [22]. In addition, performance among COD tests and volleyball-play have been previously shown to be strongly associated with sprint ability, suggesting shared underlying qualities such as explosive power and high-speed force production [23,24]. Indeed, the explosive nature of COD tasks requires a high recruitment of type II muscle fibers, particularly in the lower limbs, and is strongly influenced by neuromuscular coordination and eccentric strength, all of which appear to be well-developed in elite volleyball athletes [10,25,26]. Additionally, the three tests involve shared movement elements with the primary performance requirements of volleyball, such as explosive accelerations, sudden decelerations, and abrupt changes in direction [2,10,26,27]. These shared characteristics likely contributed to the strong inter-test correlations observed, as the athletes were well-acclimated to the underlying physical and technical demands. Taken together, these findings support the relevance and utility of all three COD assessments as effective tools for measuring COD ability in collegiate female volleyball players.

While the very large to near perfect correlations among the three tests suggest some redundancy, each test emphasizes slightly different movement patterns that may be uniquely aligned with the specific demands of volleyball. For example, the *t*-test emphasizes forward, lateral and backward movements, similar to the multidirectional nature of volleyball play. The 5-10-5 drill focuses on rapid in-line accelerations, and 180 degree turns, which closely resemble the frequent transitions of play between offense and defense. The L-drill combines lateral and diagonal movements, further mimicking the multidirectional COD needed for adjusting to the unpredictable flow of the game. Thus, any of the three tests may be relevant for testing COD ability in female volleyball players. This may be an important consideration in settings with limited time or resources, as a single COD test may suffice for routine monitoring. However, the composite score used in this study may offer a more comprehensive representation of an athlete’s COD ability, capturing performance nuances that individual tests might miss. This approach can help coaches identify specific areas for improvement by highlighting subtle performance differences that may not be detected with a single test. Nonetheless, by implementing a variety of COD tests, coaches can ensure that performance assessments reflect the broad spectrum of movement demands seen in volleyball [2], enhancing their ability to monitor and guide training adaptations.

There are a few limitations to consider when interpreting the results of the study. First, only collegiate female volleyball players were included in the sample; therefore, results should not be generalized to other populations such as male athletes, youth, or individuals from different sports. In addition, only three COD tests were explored in the current study, which may not capture the full spectrum of COD performance or movement demands specific to volleyball. Another limitation is that while all participants were NCAA Division I athletes and thus highly trained and experienced, detailed data on years of volleyball participation or prior strength and conditioning exposure were not collected. Such factors may further influence COD performance and should be considered in future studies. Lastly, the study did not assess test–retest reliability or track changes in COD ability over time in response to training. Given these constraints, the present study should be considered exploratory, providing preliminary evidence that COD tests demonstrate strong interrelationships in a relatively small sample of Division I female volleyball players but without broad generalizability.

## 5. Conclusions

The very large to near perfect correlation coefficients observed between the *t*-test, L-drill, and 5-10-5 suggest a high degree of agreement in the assessment of COD ability in female volleyball athletes. These findings contribute to our understanding of COD testing and underscore the importance of more research to refine the application of these tests in training and performance evaluation. From a practical standpoint, the findings indicate that coaches and practitioners may select any one of these COD tests to evaluate player performance, without the need to administer multiple assessments. This may save time and reduce athlete fatigue while still providing valid information on COD ability. Furthermore, because the tests used are standardized, simple to administer, and conducted under controlled conditions, the reproducibility of the study is high, allowing these methods to be readily adopted in applied volleyball and research settings.

## Figures and Tables

**Table 1:** Descriptive statistics and COD test completion times for participants.

Variable	L/DS (*n* = 6)	OH/MB (*n* = 8)	S (*n* = 2)	Total (*n* = 16)
	Means ± SD	Means ± SD	Means ± SD	Means ± SD
Age (years)	19.67 ± 1.37	19.38 ± 1.49	18.50 ± 0.50	19.40 ± 1.50
Height (cm)	170.38 ± 8.01	181.71 ± 9.01	171.38 ± 9.32	176.19 ± 10.64
Weight (kg)	64.88 ± 8.94	75.76 ± 10.05	74.59 ± 8.05	71.54 ± 11.09
*t*-test (s)	10.79 ± 0.55	10.81 ± 0.62	11.01 ± 0.54	10.88 ± 0.66
L-drill (s)	8.61 ± 0.24	8.58 ± 0.27	8.65 ± 0.26	8.59 ± 0.31
5-10-5 (s)	4.86 ± 0.13	4.89 ± 0.12	4.92 ± 0.18	4.90 ± 0.15

L/DS = Libero/Defensive Specialist, OH/MB = Outside Hitter/Middle Blocker, S = Setter, SD = Standard Deviation, cm = centimeter, kg = kilograms.

## Data Availability

The original contributions presented in the study are included in the article, further inquiries can be directed to the corresponding author/s.

## Abbreviations

The following abbreviations are used in this manuscript:

COD	Change of direction
L	Libero
DS	Defensive Specialist
OH	Outside Hitter
MB	Middle Blocker
S	Setter
SD	Standard Deviation
cm	Centimeters
kg	Kilograms
sec	Seconds

## References

[1] Nimphius S., Callaghan S.J., Bezodis N.E., Lockie R.G. (2018). Change of direction and agility tests: Challenging our current measures of performance. Strength Cond. J..

[2] Black B. (1995). Conditioning for volleyball. Strength Cond. J..

[3] Davidson A., Trewartha G. (2008). Understanding the physiological demands of netball: A time-motion investigation. Int. J. Perform. Anal. Sport.

[4] Spencer M., Lawrence S., Rechichi C., Bishop D., Dawson B., Goodman C. (2004). Time-motion analysis of elite field hockey, with special reference to repeated-sprint activity. J. Sports Sci..

[5] Stølen T., Chamari K., Castagna C., Wisløff U. (2005). Physiology of soccer: An update. Sports Med..

[6] Gabbett T.J., Kelly J.N., Sheppard J.M. (2008). Speed, change of direction speed, and reactive agility of rugby league players. J. Strength Cond. Res..

[7] Mann J.B., Ivey P.A., Mayhew J.L., Schumacher R.M., Brechue W.F. (2016). Relationship between agility tests and short sprints: Reliability and smallest worthwhile difference in national collegiate athletic association Division-I football players. J. Strength Cond. Res..

[8] Sporis G., Jukic I., Milanovic L., Vucetic V. (2010). Reliability and factorial validity of agility tests for soccer players. J. Strength Cond. Res..

[9] Stewart P.F., Turner A.N., Miller S.C. (2014). Reliability, factorial validity, and interrelationships of five commonly used change of direction speed tests. Scand. J. Med. Sci. Sports.

[10] Pauole K., Madole K., Garhammer J., LaCourse M., Rozenek R. (2000). Reliability and validity of the T-test as a measure of agility, leg power, and leg speed in college-aged men and women. J. Strength Cond. Res..

[11] Salaj S., Markovic G. (2011). Specificity of jumping, sprinting, and quick change-of-direction motor abilities. J. Strength Cond. Res..

[12] Keoliya A.A., Ramteke S.U., Boob M.A., Somaiya K.J. (2024). Enhancing Volleyball Athlete Performance: A comprehensive review of training interventions and their impact on agility, explosive power, and strength. Cureus.

[13] Gabbett T., Georgieff B. (2007). Physiological and anthropometric characteristics of Australian junior national, state, and novice volleyball players. J. Strength Cond. Res..

[14] Sheppard J.M., Young W.B. (2006). Agility literature review: Classifications, training and testing. J. Sports Sci..

[15] Hewett T.E., Myer G.D., Ford K.R. (2006). Anterior cruciate ligament injuries in female athletes: Part 1, mechanisms and risk factors. Am. J. Sports Med..

[16] Young W.B., James R., Montgomery I. (2002). Is muscle power related to running speed with changes of direction?. J. Sports Med. Phys. Fitness.

[17] Semenick D. (1990). Tests and measurements: The T-test. Strength Cond. J..

[18] McGuigan M., Haff G., Triplett T. (2016). Administration, scoring, and interpretation of selected tests. Essentials of Strength Training and Conditioning.

[19] Mishra P., Pandey C.M., Singh U., Gupta A., Sahu C., Keshri A. (2019). descriptive statistics and normality tests for statistical data. Ann. Card. Anaesth..

[20] Hopkins W.G. (2002). A scale of magnitudes for effect statistics. A New View of Statistics.

[21] Raya M.A., Gailey R.S., Gaunaurd I.A., Jayne D.M., Campbell S.M., Gagne E., Manrique P.G., Muller D.G., Tucker C. (2013). Comparison of three agility tests with male servicemembers: Edgren Side Step Test, T-Test, and Illinois Agility Test. J. Rehabil. Res. Dev..

[22] Farrow D., Pyne D., Gabbett T. (2008). Skill and physiological demands of open and closed training drills in Australian football. Int. J. Sports Sci. Coach..

[23] Zhang Z., Jiang M., Jing Y., Li M., Li Y., Yang X. (2024). Associations between sprint mechanical properties and change of direction ability and asymmetries in COD speed performance in basketball and volleyball players. Life.

[24] Lockie R.G., Dawes J.J., Callaghan S.J. (2020). Lower-body power, linear speed, and change-of-direction speed in Division I collegiate women’s volleyball players. Biol. Sport.

[25] Spiteri T., Nimphius S., Hart N.H., Specos C., Sheppard J.M., Newton R.U. (2014). Contribution of strength characteristics to change of direction and agility performance in female basketball athletes. J. Strength Cond. Res..

[26] Falch H.N., Kristiansen E.L., Haugen M.E., Van Den Tillaar R. (2021). Association of performance in strength and plyometric tests with change of direction performance in young Female Team-Sport Athletes. J. Funct. Morphol. Kinesiol..

[27] Freitas T.T., Pereira L.A., Zabaloy S., Alcaraz P.E., Arruda A.F.S., Mercer V.P., Bishop C., Loturco I. (2023). Change-of-direction and deceleration deficits in national-team female rugby sevens players: Interrelationships and associations with speed-related performance. Int. J. Sports Physiol. Perform..

